# Editorial: Psychomotor Symptomatology in Psychiatric Illnesses

**DOI:** 10.3389/fpsyt.2015.00081

**Published:** 2015-06-01

**Authors:** Sebastian Walther, Manuel Morrens

**Affiliations:** ^1^University Hospital of Psychiatry, University of Bern, Bern, Switzerland; ^2^Collaborative Antwerp Psychiatric Research Institute, University of Antwerp, Antwerp, Belgium

**Keywords:** schizophrenia, affective disorders, ADHD, Alzheimer’s disease, autism spectrum disorders

In this research topic, we have gathered articles focusing on the psychomotor component of psychiatric disorders. Indeed, motor symptoms remain as an important dimension of psychopathology that can be assessed by objective means. Particularly, in major depressive disorder and schizophrenia, motor signs have been acknowledged from the very early descriptions (1–3). But, psychomotor abnormalities have also been demonstrated in other psychiatric disorders.

This research topic included nine original articles, four reviews, three opinion papers, and one mini-review. Catatonia has been subjected to two reviews (4, 5) and one investigation of its prevalence among acutely hospitalized patients (6). Neurological soft signs have been shown to occur in autism spectrum disorders (7), in Alzheimer’s disease (8) and have been reviewed for their predictive validity in the course of schizophrenia (9). Fine motor tasks demonstrated that motor learning was preserved in schizophrenia despite cognitive and motor impairments (10, 11). In addition, psychomotor retardation was found in depressed elderly more than in elderly without depression (12). A neuroimaging study explored the cingulate motor area in motor retardation in major depression (13). The functional neuroanatomy of motor retardation in depression was also subjected to a mini-review (14). The topography of the cerebellum has been suggested as interesting focus of study to disentangle motor and cognitive functions in schizophrenia spectrum disorders (15). Two studies using actigraphy reported on gross motor activity in the course of schizophrenia (16, 17). Finally, Gawrilow and colleagues summarized the importance of motor activity in ADHD (18).

Currently, ambiguous terminology and definitions hamper research on psychomotor phenomena. In addition, some studies focus exclusively on single signs probably missing the complete picture. Therefore, we have tried to put forward a systematic approach to study psychomotor phenomena in psychotic disorders (19). In addition, van Harten and colleagues have proposed to consider movement disorders as non-mental signs of psychotic disorders just as psychiatric symptoms are classified as non-motor signs in idiopathic movement disorders (20).

One example of ongoing debate is the current discussion on the catatonia syndrome. Depending on the criteria applied, prevalence rates differ substantially (6, 21, 22), challenging the specificity of assessment methods. Despite the fact that the syndrome is quite remarkable, there is not much of a common ground in the literature as to what catatonia should be defined as. Clearly, this ambiguity of definitions has contributed to the scarcity of descriptive and interventional studies in the catatonia syndrome.

Another important field of research is the outcome of interventions in motor symptoms. Further research needs to clarify whether the motor dimension in psychiatric disorders is properly ameliorated by treating the underlying disorder or whether specific therapeutic options are required. The former would call for generalized therapies in depression, schizophrenia, or autism. The latter would instead require searching for new therapeutic targets, such as in movement disorders known in neurology. Clearly defined psychomotor disturbances may benefit from deep brain stimulation of the subthalamic nucleus (23), pedunculopontine nucleus (24), or other targets such as the reward system (25). Likewise, non-invasive brain stimulation may become a treatment option in those psychomotor disturbances related to dysfunctions in cortical motor areas.

Taken together, clarified terminology, increased awareness, and improved assessment methods will help psychomotor symptoms to become an important objective dimension of psychopathology that is informative on underlying neuropathology and longitudinal course. These transitions in psychiatric assessment will also allow for more specialized interventions for psychomotor symptoms.

## Conflict of Interest Statement

The authors declare that the research was conducted in the absence of any commercial or financial relationships that could be construed as a potential conflict of interest.
